# Genetic Diversity and Phylogeography of *Thottapalayam thottimvirus* (*Hantaviridae*) in Asian House Shrew (*Suncus murinus*) in Eurasia

**DOI:** 10.3389/fcimb.2020.00438

**Published:** 2020-08-27

**Authors:** Fuka Kikuchi, Keita Aoki, Satoshi D. Ohdachi, Kimiyuki Tsuchiya, Masaharu Motokawa, Takamichi Jogahara, Nguyễn Trường Sơn, Saw Bawm, Kyaw San Lin, Thida Lay Thwe, Chandika D. Gamage, Marie Claudine Ranorosoa, Hasmahzaiti Omar, Ibnu Maryanto, Hitoshi Suzuki, Keiko Tanaka-Taya, Shigeru Morikawa, Tetsuya Mizutani, Motoi Suzuki, Richard Yanagihara, Satoru Arai

**Affiliations:** ^1^Infectious Disease Surveillance Center, National Institute of Infectious Diseases, Tokyo, Japan; ^2^Department of Chemistry, Faculty of Science, Tokyo University of Science, Tokyo, Japan; ^3^Research and Education Center for Prevention of Global Infectious Diseases of Animals, Tokyo University of Agriculture and Technology, Fuchu, Japan; ^4^Institute of Low Temperature Science, Hokkaido University, Sapporo, Japan; ^5^Laboratory of Bioresources, Applied Biology Co., Ltd., Tokyo, Japan; ^6^The Kyoto University Museum, Kyoto University, Kyoto, Japan; ^7^Faculty of Law, Economics and Management, Okinawa University, Naha, Japan; ^8^Institute of Ecology and Biological Resources, Vietnam Academy of Science and Technology, Hanoi, Vietnam; ^9^Graduate University of Science and Technology, Vietnam Academy of Science and Technology, Hanoi, Vietnam; ^10^Department of Pharmacology and Parasitology, University of Veterinary Science, Nay Pyi Taw, Myanmar; ^11^Department of Aquaculture and Aquatic Disease, University of Veterinary Science, Nay Pyi Taw, Myanmar; ^12^Department of Zoology, Yangon University of Distance Education, Yangon, Myanmar; ^13^Department of Microbiology, Faculty of Medicine, University of Peradeniya, Peradeniya, Sri Lanka; ^14^Mention Foresterie et Environnement, Ecole Supérieur des Sciences Agronomiques, Université d'Antananarivo, Antananarivo, Madagascar; ^15^Institute of Biological Sciences, Faculty of Science, University of Malaya, Kuala Lumpur, Malaysia; ^16^Research Centre for Biology, Indonesian Institute of Sciences (LIPI), Bogor, Indonesia; ^17^Laboratory of Ecology and Genetics, Graduate School of Environmental Science, Hokkaido University, Sapporo, Japan; ^18^Department of Microbiology, Faculty of Veterinary Medicine, Okayama University of Science, Imabari, Japan; ^19^Pacific Center for Emerging Infectious Diseases Research, John A. Burns School of Medicine, University of Hawaii at Manoa, Honolulu, HI, United States

**Keywords:** *Thottapalayam thottimvirus*, *Suncus murinus*, genetic diversity, phylogeography, shrew-borne hantavirus

## Abstract

Murid and cricetid rodents were previously believed to be the principal reservoir hosts of hantaviruses. Recently, however, multiple newfound hantaviruses have been discovered in shrews, moles, and bats, suggesting a complex evolutionary history. Little is known about the genetic diversity and geographic distribution of the prototype shrew-borne hantavirus, *Thottapalayam thottimvirus* (TPMV), carried by the Asian house shrew (*Suncus murinus*), which is widespread in Asia, Africa, and the Middle East. Comparison of TPMV genomic sequences from two Asian house shrews captured in Myanmar and Pakistan with TPMV strains in GenBank revealed that the Myanmar TPMV strain (H2763) was closely related to the prototype TPMV strain (VRC66412) from India. In the L-segment tree, on the other hand, the Pakistan TPMV strain (PK3629) appeared to be the most divergent, followed by TPMV strains from Nepal, then the Indian-Myanmar strains, and finally TPMV strains from China. The Myanmar strain of TPMV showed sequence similarity of 79.3–96.1% at the nucleotide level, but the deduced amino acid sequences showed a high degree of conservation of more than 94% with TPMV strains from Nepal, India, Pakistan, and China. Cophylogenetic analysis of host cytochrome *b* and TPMV strains suggested that the Pakistan TPMV strain was mismatched. Phylogenetic trees, based on host cytochrome *b* and cytochrome c oxidase subunit I genes of mitochondrial DNA, and on host recombination activating gene 1 of nuclear DNA, suggested that the Asian house shrew and Asian highland shrew (*Suncus montanus*) comprised a species complex. Overall, the geographic-specific clustering of TPMV strains in Asian countries suggested local host-specific adaptation. Additional in-depth studies are warranted to ascertain if TPMV originated in Asian house shrews on the Indian subcontinent.

## Introduction

More than four decades following the original isolation of *Thottapalayam thottimvirus* (TPMV) from an Asian house shrew (*Suncus murinus*) in southern India (Carey et al., 1971), other genetically distinct hantaviruses (family *Hantaviridae*) have been detected in shrews (order Eulipotyphla, family Soricidae) of multiple species in Europe, Asia, Africa, and North America. To date, these include Imjin thottimvirus in the Ussuri white-toothed shrew (*Crocidura lasiura*) (Song et al., 2009), Kilimanjaro thottimvirus in the Kilimanjaro mouse shrew (*Myosorex zinki*) (Kang et al., 2014), Uluguru thottimvirus in the geata mouse shrew (*Myosorex geata*) (Kang et al., 2014), Tanganya orthohantavirus in the Therese's shrew (*Crocidura theresae*) (Klempa et al., 2007), Azagny orthohantavirus in the West African pygmy shrew (*Crocidura obscurior*) (Kang et al., 2011b), Jeju orthohantavirus in the Asian lesser white-toothed shrew (*Crocidura shantungensis*) (Arai et al., 2012), Bowé orthohantavirus in the Doucet's musk shrew (*Crocidura douceti*) (Gu et al., 2013), Cao Bǎng orthohantavirus in the Chinese mole shrew (*Anourosorex squamipes*) (Song et al., 2007c), Seewis orthohantavirus in the Eurasian shrew (*Sorex araneus*) (Song et al., 2007b), Ash River orthohantavirus in the masked shrew (*Sorex cinereus*) (Arai et al., 2008), Jemez Springs orthohantavirus in the dusky shrew (*Sorex monticolus*) (Arai et al., 2008), Kenkeme orthohantavirus in the flat-skulled shrew (*Sorex roboratus*) (Kang et al., 2010), Asikkala orthohantavirus in the Eurasian pygmy shrew (*Sorex minutus*) (Radosa et al., 2013), Yákèshí orthohantavirus in the taiga shrew (*Sorex isodon*) (Guo et al., 2013), Quan Hu Shan orthohantavirus in the greater stripe-backed shrew (*Sorex cylindricauda*) (Zuo et al., 2014), Boginia orthohantavirus in the Eurasian water shrew (*Neomys fodiens*) (Gu et al., 2013), and Camp Ripley orthohantavirus in the northern short-tailed shrew (*Blarina brevicauda*) (Arai et al., 2007). Data suggest that shrews, rather than rodents, may have served as the earlier mammalian hosts of hantaviruses (Song et al., 2007a; Kang et al., 2011a; Bennett et al., 2014; Yanagihara et al., 2014). That said, the evolutionary history of hantavirus expansion is still unclear. In particular, the genetic diversity and phylogeography of the prototype shrew-borne hantavirus (TPMV) warrants clarification.

## Materials and Methods

### Ethics Statement

The guidelines of the American Society of Mammalogists (Kirkland, 1998; Sikes and Animal Care and Use Committee of the American Society of Mammalogists, 2016) were followed for trapping and euthanasia of shrews and for tissue collection and processing. And approvals were obtained from the Ministry of Agriculture and Rural Development in Vietnam and the Institutional Animal Care and Use Committee of the National Institute of Infectious Diseases to conduct the study (permission numbers: 108074, 111126, 112152, 115162, 118180).

### Animals

**Figure 1:**
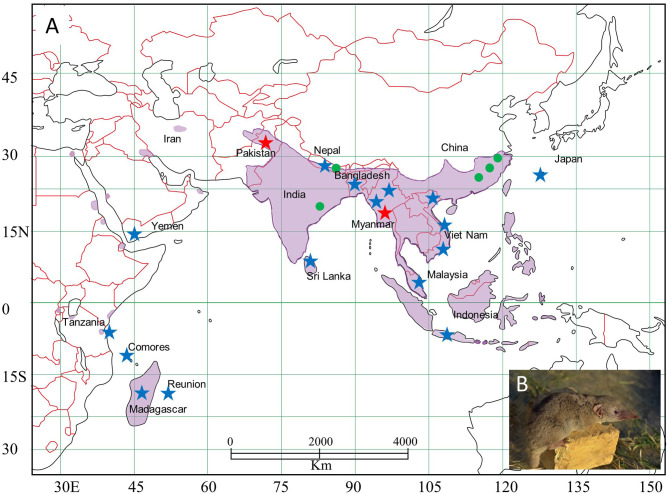
Map, showing the distribution of Asian house shrews (*Suncus murinus*) in Eurasia. **(A)** Asian house shrews were captured in multiple locations (indicated by star). TPMV-infected shrews were found in Pakistan (red star), Myanmar (red star), India (green circle), Nepal (green circle), and China (green circle). **(B)** Photo of Asian house shrew.

Asian house shrews were collected, using Sherman live traps, during biological distribution surveys from 2011 to 2016 in Asia (Indonesia, Japan, Malaysia, Myanmar, Pakistan, Sri Lanka, Vietnam, Yemen) and Africa (Comoros, Madagascar, Tanzania) (Ohdachi et al., 2016) (Figure 1). Lung tissues, preserved in RNA*later*® Stabilization Solution, were analyzed for hantavirus RNA by reverse transcription polymerase chain reaction (RT-PCR) (Arai et al., 2016a). Asian house shrews from Bangladesh (early-onset diabetes in *Suncus*: EDS) and Nepal (Kathmandu: KAT), which were being maintained for experimentation, were also used for host genetic analysis (Jogahara, 2016) (Supplementary Data S1).

### RNA Extraction and cDNA Synthesis

Total RNA was extracted from RNA*later*®-preserved lung tissues, using the GC series Magtration®-MagaZorb® RNA Common N Kit or MagDEA RNA 100 Kit (Precision System Science, Matsudo, Japan), and then reverse transcribed, using PrimeScript™ II 1st strand cDNA Synthesis Kit (Takara Bio, Otsu, Japan) and oligonucleotide primer (OSM55F, 5′-TAGTAGTAGACTCC−3′), designed from the conserved 5′-ends of the S, M, and L segments of hantaviruses (Klempa et al., 2006, 2007; Song et al., 2007c; Arai et al., 2008, 2016a).

### RT-PCR and DNA Sequencing

Nested primers for TPMV and other recently identified shrew-borne hantaviruses were used to initially screen tissues for hantavirus RNA (Song et al., 2009; Kang et al., 2011c; Gu et al., 2013). Thereafter, amplification of the full-length S-, M-, and L-genomic segments was attempted. Oligonucleotide primer sequences have been deposited as Supplementary Data S2. First- and second-round PCR was performed in 20-μL reaction mixtures, containing 250 μM dNTP, 2 mM MgCl_2_, and 0.25 μM of each primer. LA Taq hot start version (Takara Bio) and AmpliTaq gold 360 DNA polymerase (Applied Biosystems, Foster City, CA, USA) were used at 1 U each for the first- and second-round PCR, respectively (Arai et al., 2016b). Initial denaturation at 94°C for 2 min was followed by two cycles each of denaturation at 94°C for 30 s, two-degree step-down annealing from 48 to 38°C for 40 s, and elongation at 68°C for 1 min, then 32 cycles of denaturation at 94°C for 40 s, annealing at 42°C for 40 s, and elongation at 68°C for 1 min, in a Veriti thermal cycler (Applied Biosystems) and Mastercycler X50 (Eppendorf, Hamburg, Germany) (Arai et al., 2008, 2012). Amplicons were treated with Exonuclease I and Shrimp Alkaline Phosphatase (New England Biolabs, Ipswich, MA, USA) for 30 min. DNA was sequenced directly using an ABI Prism 377XL Genetic Analyzer (Applied Biosystems) (Arai et al., 2007; Kang et al., 2011c).

### Genetic and Phylogenetic Analysis

Partial S-, M-, and L-segment nucleotide and amino acid sequences, amplified from Asian house shrews, were aligned with available hantavirus sequences, using the ClustalW in BioEdit (Thompson et al., 1994). The degree of sequence homology was assessed by pair-wise comparisons (Kang et al., 2009, 2011a). Phylogenetic trees were constructed using MrBayes 3.1.2 (Ronquist and Huelsenbeck, 2003), with the GTR+I+*Γ* model of evolution, as selected by using jModelTest version 2.1.7 (Darriba et al., 2012). Bayesian analysis consisted of 10 million Markov chain Monte Carlo generations to ensure convergence across two runs of six chains each, with average standard deviations of split frequencies <0.01 and effective sample sizes well over 100, resulting in consensus trees supported by posterior-node probabilities (Kang et al., 2009, 2011a). The co-evolutionary relationships between hantaviruses and their shrew and rodent reservoir hosts were analyzed by the comparative concordance between host and hantavirus cladograms in TreeMap 3b1243 (Charleston and Robertson, 2002; Kang et al., 2009; Arai et al., 2012).

### mtDNA and Nuclear Genes Sequencing and Host Phylogeny

To verify the geographic diversity of Asian house shrews and to study their phylogenetic relationships, genomic DNA was extracted from lung tissue using the MagDEA® DNA 200 (GC) (Precision System Science). The entire 1,140-nucleotide cytochrome *b* (cyt*b*) gene of mitochondrial DNA (mtDNA), the 1,545-nucleotide cytochrome c oxidase subunit I *(COI)* gene and the recombination activating gene 1 *(RAG1)* were amplified using the following primer sets: Cy-14724F (5′-GACYARTRRCATGAAAAAYCAYCGTTGT−3′)/Cy-15909R (5′-CYYCWTYIYTGGTTTA CAAGACYAG−3′) (Arai et al., 2016b) and KOD multi-enzyme (Toyobo, Osaka, Japan), MammMt-5533F (5′-CYCTGTSYTTRRATTTACAGTYYAA−3′)/MammMt-7159R (5′-GRGGTTCRAWW CCTYCCTYTCTT−3′) (Arai et al., 2019) and Phusion enzyme (New England Biolabs), and newly designed primers RAG1-61F (5′-TCTGCACCYGATGAAATTCARCACC−3′)/RAG1-3139R (5′-CTCCATTGAATCTTGGCTTTCC−3′) and KOD multi-enzyme, respectively. PCR was performed in 50-μL reaction mixtures, containing 200 μM dNTP and 1 U of KOD multi and Epi DNA polymerase or Phusion enzyme. Initial denaturation was at 95°C for 2 min, followed by two cycles each of denaturation at 95°C for 15 s, two-degree step-down annealing from 60 to 50°C for 30 s, and elongation at 68°C for 1 min 30 s, then 30 cycles of denaturation at 95°C for 15 s, annealing at 55°C for 30 s, and elongation at 68°C for 1 min 30 s, in a Veriti thermal cycler (Arai et al., 2019). PCR products were purified by Mobispin S-400 (Molecular Biotechnology, Lotzzestrasse, Germany) and were sequenced directly (Arai et al., 2012, 2019). The models of host nucleotide evolution were selected under jModeltest version 2.1.7, the GTR+I+*Γ* model for host phylogenetic sequence set, the TrN+G for Cyt*b*, TIM3+ I for *COI* of and TPM2uf +I models for *RAG1* of *Suncus* sequence sets. The results of modeltest were shown in Supplementary Data 3A–G. Host phylogenetic analysis also consisted of 10 million Markov chain Monte Carlo generations to ensure convergence across two runs of six chains each, with average standard deviations of split frequencies <0.01 and effective sample sizes well over 100, resulting in consensus trees supported by posterior-node probabilities.

## Results

### Hantavirus Detection

In all but two of the 198 shrew lung tissue samples, multiple attempts to detect hantavirus RNA were unsuccessful (Table 1). The exceptions were one of 11 and one of three Asian house shrews from Pakistan (captured in Karachi: 24.947802 N, 67.122999 E) and Myanmar (captured near a cattle farm in Taung gyi, Shan state: 20.804169 N, 97.060360 E, detail in Supplementary Table 1), respectively, collected in 2013. Sequence analysis of the amplicons revealed TPMV. Amplification of the full-coding region of the S segment and the partial M and L segments was achieved for TPMV strain H2763 (Myanmar), while only partial L-segment sequences were obtained for TPMV strain PK3629 (Pakistan).

**Table 1:** RT-PCR detection of TPMV RNA in Asian house shrews.

**Country**	**TPMV RNA positive/shrews tested**						
	**2011**	**2012**	**2013**	**2014**	**2015**	**2016**	**Total**
Comoros		0/3					0/3
Indonesia					0/6		0/6
Japan	0/24	0/5	0/3		0/1	0/18	0/51
Madagascar				0/11	0/18		0/29
Malaysia			0/8				0/8
Myanmar		0/3	1/11	0/6	0/3	0/2	1/25
Pakistan			1/11				1/11
Réunion		0/4					0/4
Sri Lanka				0/10			0/10
Tanzania				0/7			0/7
Vietnam		0/18	0/18				0/36
Yemen					0/8		0/8
	0/24	0/33	2/51	0/34	0/36	0/20	2/198

### Nucleotide and Amino Acid Sequence Analysis

Analysis of the S-, M-, and L-segment sequences of TPMV strain H2763 from Myanmar indicated an overall genomic organization similar to prototype TPMV strain VRC66412 from India. The 1,506-nucleotide S-genomic segment encoded a nucleocapsid (N) protein of 435 amino acids, possibly starting at nucleotide position 68, and a 130-nucleotide 3′-non-coding region. The TPMV S-genomic segment, like that of other recently described hantaviruses in shrews, did not contain the hypothetical NSs open reading frame, typically found in hantaviruses harbored by cricetid rodents.

### Hantavirus Phylogeny

**Figure 2:**
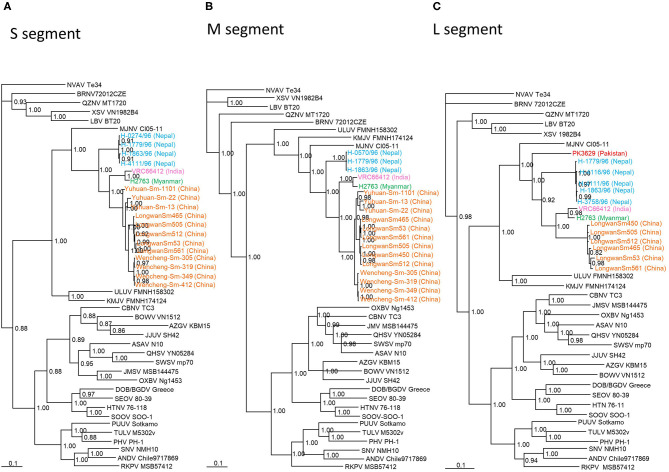
Phylogenetic trees generated by the Bayesian method, under the best-fit GTR+I+*Γ* model of evolution, based on the partial S-, M-, and L-genomic segments of TPMV strains H2763 (S: MT225396; M: MT225397; L: MT225398) in Myanmar and PK3629 in Pakistan (L: MT225399), and other representative hantaviruses. The phylogenetic positions of TPMV strains VRC66412 (S: AY526097, M: EU001329, L: EU001330) in India; TPMV strains LongwanSm53 (S: JF784172; M: JF784178; L: KJ420575), LongwanSm450 (S: JF784173, M: JF784179, L: KJ420576), LongwanSm465 (S: JF784174, M: JF784180), LongwanSm505 (S: JF784175, M: JF784181), LongwanSm512 (S: JF784176, M: JF784182), LongwanSm561 (S: JF784177, M: JF784183), Wencheng-Sm-305 (S: KJ420560, M: KJ420542), Wencheng-Sm-319 (S: KJ420561, M: KJ420543, L: KJ420569), Wencheng-Sm-349 (S: KJ420562, M: KJ420544, L: KJ420570), Wencheng-Sm-412 (S: KJ420563, M: KJ420545, L: KJ420571), Yuhuan-Sm-13 (S: KJ420564, M: KJ420546, L: KJ420572), Yuhuan-Sm-22 (S: KJ420565, M: KJ420547, L: KJ420573), and Yuhuan-Sm-1101 (S: KJ420566, M: KJ420548, L: KJ420574) in China; strains H-1091/96 (S: HQ831358), H-0274/96 (S: HQ831359), H-0570/96 (S: HQ831360, M: HQ831376), H-0898/96 (S: HQ831361), H-1779/96 (S: HQ831362, M: HQ831375, L: HQ831374), H-1863/96 (S: HQ831363, M: HQ831377, L: HQ831373), H-3753/96 (S: HQ831364), H-3758/96 (S: HQ831365, L: HQ831372), H-3858/96 (S: HQ831366), H-4066/96 (S: HQ831367), H-4111/96 (S: HQ831368, L: HQ831371), and H-4116/96 (S: HQ831369, L: HQ831370) in Nepal are shown in relationship to Imjin thottimvirus (MJNV Cl05-11, S: EF641804; M: EF641798; L: EF641806) from *Crocidura lasiura*, Uluguru thottimvirus (ULUV FMNH158302, S: JX193695; M: JX193696; L: JX193697) from *Myosorex geata*, Kilimanjaro thottimvirus (KMJV FMNH174124, S: JX193698; M: JX193699; L: JX193700) from *Myosorex zinki*, Jeju orthohantavirus (JJUV SH42, S: HQ663933; M: HQ663934; L: HQ663935) from *Crocidura shantungensis*, Oxbow orthohantavirus (OXBV Ng1453, S: FJ5339166; M: FJ539167; L: FJ593497) from *Neurotrichus gibbsii*, Cao Bǎng orthohantavirus (CBNV TC-3, S: EF543524; M: EF543526; L: EF543525) from *Anourosorex squamipes*, Azagny orthohantavirus (AZGV KBM15, S: JF276226; M: JF276227; L: JF276228) from *Crocidura obscurior*, Bowé orthohantavirus (BOWV VN1512, S: KC631782; M: KC631783; L: KC631784) from *Crocidura douceti*, Qian Hu Shan orthohantavirus (QHSV YN05-284, S: GU566023; M: GU566022; L:GU566021) from *Sorex cylindricauda*, Seewis orthohantavirus (SWSV mp70, S: EF636024; M: EF636025; L: EF636026) from *Sorex araneus*, Jemez Springs orthohantavirus from *Sorex monticolus* (JMSV MSB144475, S: FJ593499; M: FJ593500; L: FJ593501), Asama orthohantavirus (ASAV N10, S: EU929072; M: EU929075; L: EU929078) from *Urotrichus talpoides*, Nova mobatvirus (NVA Te34, S: KR072621, M: KR072622, L: KR072623) from *Talpa europaea*, and Rockport orthohantavirus (RKPV MSB57412, S: HM015223; M: HM015222; L: HM015221) from *Scalopus aquaticus*. Also shown are representative rodent-borne hantaviruses, including Sin Nombre orthohantavirus (SNV NMH10, S: NC_005216; M: NC_005215; L: NC_005217), Andes orthohantavirus (ANDV Chile9717869, S: AF291702; M: AF291703; L: AF291704), Prospect Hill orthohantavirus (PHV PH-1, S: Z49098; M: X55129; L: EF646763), Tula orthohantavirus (TULV M5302v, S: NC_005227; M: NC_005228; L: NC_005226), Puumala orthohantavirus (PUUV Sotkamo, S: NC_005224; M: NC_005223; L: NC_005225), Dobrava/Belgrade orthohantavirus (DOB/BGDV Greece, S: NC_005233; M: NC_005234; L: NC_005235), Hantaan orthohantavirus (HTNV 76-118, S: NC_005218; M: NC_005219; L: NC_005222) and Seoul orthohantavirus (SEOV 80-39, S: NC_005236; M: NC_005237; L: NC_005238), and bat-borne hantaviruses, Brno loanvirus (BRNV 7/2012/CZE, S: KX845678; M: KX845679; L: KX845680) from *Nyctalus noctula*, Láibin mobatvirus (LAIV BT20, S: KM102247; M: KM102248; L: KM102249) from *Taphozous melanopogon*, Xuân Son mobatvirus (XSV VN1982B4, S: KC688335; L: JX912953) from *Hipposideros pomona* and Quezon mobatvirus (QZNV MT1720/1657, S: KU950713; M: KU950714; L: KU950715) from *Rousettus amplexicaudatus*, respectively. The numbers at each node are posterior node probabilities based on 45,000 trees: two replicate Markov chain Monte Carlo runs consisting of six chains of two million generations each sampled every 100 generations with a burn-in of 7,500 (25%). The scale bar indicates nucleotide substitutions per site. **(A)** S-segment, **(B)** M-segment, **(C)** L-segment phylogenetic trees. Color of TPMV strains: red (Pakistan); orange (China); green (Myanmar); light blue (Nepal); pink (India).

TPMV strain H2763 from Myanmar appeared as one cluster in phylogenetic trees, based on the S-, M-, and L-segment sequences, using the Bayesian methods (Figure 2). The TPMV strain PK3629 from Pakistan also constructed one cluster in a tree based on the L segment. The phylogenetic trees suggested that the primordial strain of TPMV originated in northern India and surrounding countries, including Pakistan or Nepal (Figure 2).

### Pair-Wise Alignment and Comparison

Pair-wise alignment and comparison of the S segment (1,506 nucleotides), M segment (2,382 nucleotides), and L segment (4,963nucleotides) revealed that TPMV strain H2763 from Myanmar exhibited high sequence similarity to prototype TPMV strain VRC66412 from southern India. The TPMV strain PK3629 from Pakistan showed low nucleotide sequence similarity (79.3%) in the L segment, but the encoded amino acid sequences were highly conserved (94.1–99.2%) with TPMV strains from Nepal, India, Pakistan, and China (Table 2). Compared with representative hantaviruses from rodents, shrews, and bats, the TPMV strain from Myanmar differed by ~20–60% at the nucleotide and amino acid levels for each segment.

**Table 2:** Nucleotide and amino acid sequence similarity (%) between TPMV strain H2763 and other TPMV strains, as well as representative rodent-, shrew-, and bat-borne hantaviruses.

**•**	**S-segment**	**NP**	**M-segment**	**GP**	**L-segment**	**RdRp**
**Hantavirus strain**	**1308 nt**	**435 aa**	**2188 nt**	**240 aa**	**4963 nt**	**1654 aa**
TPMV VRC66412	94.7%	98.4%	96.1%	99.2%	94.9%	98.4%
TPMV H0274/96	80.0%	98.2%	–	–	–	–
TPMV H0570/96	–	–	81.7%	94.8%	–	–
TPMV H1779/96	81.1%	97.8%	81.7%	95.2%	81.3%	96.7%
TPMV H1863/96	80.6%	97.7%	81.5%	94.7%	80.2%	95.9%
TPMV H3758/96	–	–	–	–	81.2%	96.4%
TPMV H4111/96	80.6%	98.0%	–	–	80.5%	96.0%
TPMV H4116/96	–	–	–	–	80.4%	95.8%
TPMV Longwan53	84.0%	97.2%	84.0%	96.4%	82.2%	96.4%
TPMV Longwan450	84.0%	97.9%	84.1%	95.9%	82.7%	95.5%
TPMV Longwan465	84.5%	98.8%	84.1%	95.3%	82.8%	96.3%
TPMV Longwan505	84.4%	98.6%	84.1%	96.2%	83.2%	96.3%
TPMV Longwan512	84.4%	98.8%	83.9%	95.7%	83.0%	96.4%
TPMV Longwan561	84.3%	98.8%	84.1%	95.3%	82.5%	96.4%
TPMV Wencheng305	84.3%	98.6%	84.3%	96.2%	–	–
TPMV Wencheng319	84.0%	98.4%	84.3%	96.6%	–	–
TPMV Wencheng349	84.3%	98.1%	84.4%	96.6%	–	–
TPMV Wencheng412	84.5%	98.8%	84.3%	96.6%	–	–
TPMV Yuhuan1101	–	–	84.1%	96.6%	–	–
TPMV PK3629	–	–	–	–	79.3%	94.1%
HTNV 76-118	55.0%	48.1%	56.5%	51.7%	62.0%	62.5%
DOB/BGDV Greece	55.1%	47.6%	56.9%	52.6%	62.3%	62.1%
SEOV HR80-39	54.8%	46.7%	57.4%	51.0%	61.7%	61.9%
SOOV SOO-1	56.0%	49.1%	57.5%	51.8%	62.4%	62.4%
PUUV Sotkamo	55.5%	46.0%	57.1%	50.8%	62.7%	61.9%
TULV M5302v	54.6%	45.8%	57.4%	50.6%	62.4%	61.5%
PHV PH-1	55.0%	46.9%	56.8%	50.8%	61.1%	61.2%
SNV NMH10	55.0%	48.8%	56.3%	50.6%	62.0%	62.0%
ANDV Chile9717869	56.9%	48.4%	58.5%	51.9%	61.6%	61.9%
CBNV CBN-3	56.9%	48.9%	57.5%	52.3%	62.1%	62.8%
QHSV YN05-284	52.7%	44.1%	57.2%	51.2%	69.4%	71.8%
SWSV mp70	51.9%	44.6%	61.7%	57.8%	59.5%	58.0%
JMSV MSB144475	54.7%	47.9%	57.0%	48.8%	61.5%	61.4%
AZGV KBM15	55.5%	46.0%	53.3%	42.5%	61.1%	61.5%
BOWV VN1512	54.1%	47.5%	55.9%	48.6%	62.1%	62.1%
JJUV SH42	54.8%	46.3%	56.0%	50.6%	62.5%	61.7%
MJNV Cl05-11	66.7%	71.3%	70.8%	77.1%	73.8%	80.6%
ULUV FMNH158302	60.9%	62.5%	60.2%	51.6%	69.1%	74.0%
KMJV FMNH174124	66.5%	68.0%	65.1%	65.9%	69.1%	74.2%
NVAV Te34	53.9%	47.4%	58.2%	51.2%	64.8%	63.8%
RKPV MSB57412	54.5%	48.6%	56.6%	50.6%	61.7%	61.2%
ASAV N10	55.3%	45.9%	58.3%	51.0%	62.8%	62.8%
OXBV Ng1453	52.7%	48.2%	56.1%	49.7%	61.5%	61.3%
XSV VN1982B4	54.3%	45.8%	55.4%	49.2%	64.0%	65.0%
LAIV BT20	54.3%	44.6%	56.8%	50.5%	64.6%	66.2%
QZNV MT1720/1657	55.1%	45.8%	57.3%	51.2%	62.9%	64.6%
BRNV 7/2012/CZE	54.6%	47.0%	56.0%	47.4%	63.4%	63.2%

*Dobrava-Belgrade orthohantavirus (DOB/BGDV) Greece, Hantaan orthohantavirus (HTNV) 76-118, Seoul orthohantavirus (SEOV) HR80-39 and Soochong orthohantavirus (SOOV) SOO-1 were detected in murid rodents; Puumala orthohantavirus (PUUV) Sotkamo, Prospect Hill orthohantavirus (PHV) PH-1, Tula orthohantavirus (TULV) M5302v, Sin Nombre orthohantavirus (SNV) NMH10 and Andes orthohantavirus (ANDV) Chile9717869 in cricetid rodents; Azagny orthohantavirus (AZGV) KBM15, Bowé orthohantavirus (BOWV) VN1512, Jeju orthohantavirus (JJUV) SH42, Imjin thottimvirus (MJNV) Cl05-11 and Thottapalayam thottimvirus (TPMV) VRC66412 in crocidurine shrews; Cao Bǎng orthohantavirus (CBNV) CBN-3, Seewis orthohantavirus (SWSV) mp70, Jemez Springs orthohantavirus (JMSV) MSB144475, Qian Hu Shan orthohantavirus (QHSV) YN05-284 in soricine shrews (orange); Uluguru thottimvirus (ULUV) FMN158302 and Kilimanjaro thottimvirus (KMJV) FMNH174124 in myosoricine shrews; Rockport orthohantavirus (RKPV) MSB57412, Oxbow orthohantavirus (OXBV) Ng1453, Asama orthohantavirus (ASAV) N10 and Nova mobatvirus (NVAV) Te34 in moles (light blue); and Xuân Son mobatvirus (XSV) VN1982B4, Láibin mobatvirus (LAIV) BT20, Quezon mobatvirus (QZNV) MT1720/1657 and Brno loanvirus (BRNV) 7/2012/CZE in bats (light green). Nucleocapsid protein (NP), Glycoprotein (GP) and RNA-dependent RNA polymerase (RdRp) are shown in the header. Blue color indicates AZGV, BOWV and JJUV in genus Orthohantavirus from crocidurine shrews, and Yellow color indicates TPMVs. The bolded numbers show nucleotides and amino acids*.

### Co-phylogenetic Analysis of Asian House Shrew and TPMV

**Figure 3:**
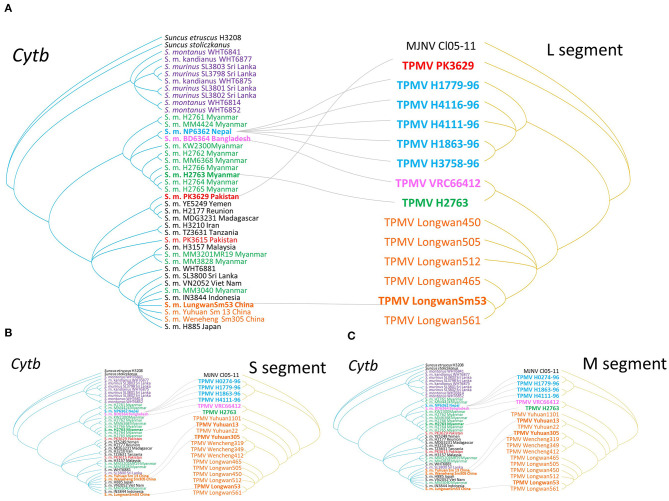
Comparisons of phylogenies of TPMV and their host species Asian house shrew. The host trees on the left were based on cytochrome *b* (cyt*b*) gene, while the hantavirus tree on the right was based on the nucleotide sequences of L- **(A)**, S- **(B)**, and M-segments **(C)**, respectively. Letterings for taxa are shown in purple for Sri Lanka, green for Myanmar, blue for Nepal, pink for Bangladesh and India, red for Pakistan, orange for China, black for the other countries, and out groups in both trees. Nepal Asian house shrew was adapted experimental animal strain NP6362. Bangladesh experimental strain BD6364 was used for alternative Indian strain.

As evidenced by co-phylogeny mapping, using a consensus tree based on L-segment sequences, TPMV strains segregated according to the geographic locations of the Asian house shrews (Figures 3B,C). The phylogenetic positions of TPMV strains based on the S and M segments mirrored the phylogenetic relationships of their Asian house shrews, except for the Pakistan strain in the L-segment tree. The Pakistan strain was mismatched between virus and host phylogeography (Figure 3A).

### Phylogenetic Analysis of Asian House Shrew

**Figure 4:**
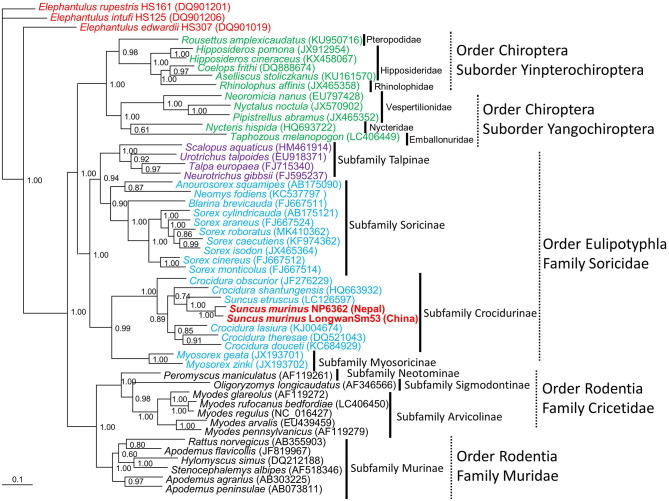
Bayesian phylogenetic tree, based on the 1,140-nucleotide cytochrome *b* gene of mtDNA of small mammals within the order Eulipotyphla (families Talpidae and Soricidae), order Rodentia (families Muridae and Cricetidae) and order Chiroptera, suborder Yinpterochiroptera (families Pteropodidae, Hipposideridae, Rhinolophidae), and suborder Yangochiroptera (families Nycteridae, Emballonuridae and Vespertilionidae). The tree was rooted using Elephantulus (order Macroscelidea, GenBank accession numbers DQ901019, DQ901206, and DQ901201) as the outgroup. Numbers at nodes indicate posterior probability values (>0.7) based on 150,000 trees: two replicate Markov chain Monte Carlo runs, consisting of six chains of 10 million generations each sampled every 100 generations with a burn-in of 25,000 (25%). Scale bars indicate nucleotide substitutions per site. Letterings for taxa are shown in green for bats, blue for shrews, purple for moles, black for rodents, red for Elephantulus, and red bold for Asian house shrew. The GenBank accession number for the cyt*b* sequence for Asian house shrews are MT344840 in Nepal and JF784169 in China.

The molecular identification of TPMV-infected shrews was confirmed as *S. murinus murinus* by amplification and sequencing of the cyt*b* and *COI* genes of mtDNA and *RAG1* gene of nuclear DNA. Phylogenetic analysis based on the cyt*b* gene indicated Asian house shrews and Etruscan shrews (*Suncus etruscus*) were clearly distinct (Figure 4). However, the relationships between Asian house shrews, Asian highland shrews (*Suncus montanus*) and, some *S. murinus* subspecies (such as *S. m. murinus, S. m. kandianus*, and *S. m. caerulescens*) (Meegaskumbura et al., 2010) were less clear.

**Figure 5:**
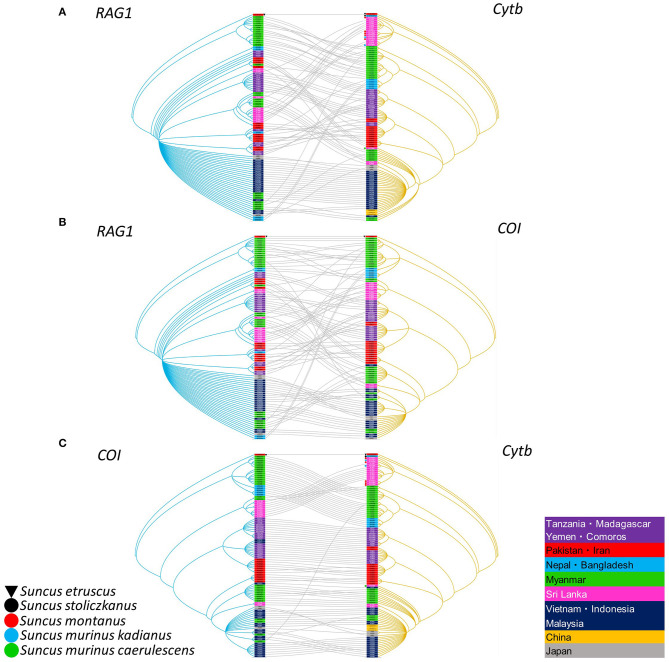
Comparisons of nuclear and mitochondria genes generated by TreeMap 3, using Bayesian method, based on the recombination activating gene 1 *(RAG1)*, cytochrome *b* (cyt*b*) gene, and cytochrome oxidase I *(COI)* gene of the Asian house shrew and related shrews. **(A)** Left tree on the *RAG1* sequence, while the mitochondria tree on the right was based on the *COI* gene or **(B)** cyt*b* gene, and **(C)** also comparison of cyt*b* and *COI* genes, respectively. The nuclear (*RAG-1*) and mitochondria relationship (cyt*b* and *COI*) were listed in Supplementary Table 1. Letterings for taxa are shown in right table. The deposited cyt*b* sequences are attached dot. The tree was rooted using *Suncus etruscus* (order Eulipotyphla, family Soricidae, GenBank accession numbers: cyt*b*, LC126597; *COI*, MK410384; and *RAG1*, MT344767). The *RAG1* order in Figures 5A,B was the same. The order of *COI* and cyt*b* in Figure 5C was adjusted for optimal.

Segregation of Asian house shrews in Asia and Africa was demonstrated by co-phylogeny mapping, using consensus trees based on the cyt*b, COI* and *RAG1* genes (Figure 5). The phylogenetic positions of *RAG1* in nuclear DNA and cyt*b* and *COI* in mtDNA were not synchronized for each gene. These data suggest that *S. murinus* and *S. montanus* are hybrid species and comprise the *S. murinus*-*S. montanus* species complex.

## Discussion

The Asian house shrew, one of 18 species in the genus *Suncus*, is widely distributed throughout Asia and the Pacific, Africa, and the Middle East (Figure 1). It is peridomestic, typically found within areas of human habitation, and has become dependent on discarded human food waste. Asian house shrews may have been intentionally introduced by humans, similar to *Rattus* rodents, into Africa (Egypt, Eritrea, Kenya, Republic of Djibouti, Rwanda, Sudan, and Tanzania), the Middle East (Iran, Iraq, Kingdom of Bahrain, Kuwait, Saudi Arabia, Sultanate of Oman, and Yemen), the islands within the Indian Ocean (Comoros, Republic of Madagascar, Republic of Mauritius, and Réunion), and Asia and the Pacific (Japan, Guam, and Philippines) (Kang et al., 2011c). Genetic analysis and treemap dendrograms of *RAG1* and *COI, RAG1* and cyt*b*, and *COI* and cyt*b* suggest that Asian house shrews may represent hybrids with the Asian highland shrew in Sri Lanka and some area of Eurasia (Figure 5).

The previously held conventional view that hantaviruses co-evolved with their reservoir hosts has been challenged recently by the conjecture that preferential host switching and local host-specific adaptation account for the congruent phylogenies of hantaviruses and their small mammal hosts (Ramsden et al., 2009). Multiple examples of host sharing are now known for hantaviruses hosted by rodents and shrews (Yanagihara et al., 2014). Whether or not TPMV exhibits such host sharing with evidence of carriage by other species of the genus *Suncus* requires future investigation.

Based on phylogenetic analysis of mtDNA and nuclear genes, as well as karyotype and morphological analysis, the taxonomy of the Asian house shrew is still unclear. Asian house shrews include at least two subspecies (*S. murinus murinus, S. murinus kandianus*, and *S. murinus caerulescens*), and the Asian highland shrew (*S. montanus*) is morphologically very similar. Our genetic analysis suggests that morphological based *S. m. murinus, S. m. kadianus*, S. m. caerulescens, and *S. montanus* represent hybrid species. Thus, a species complex has been proposed (Ohdachi et al., 2016).

**Figure 6:**
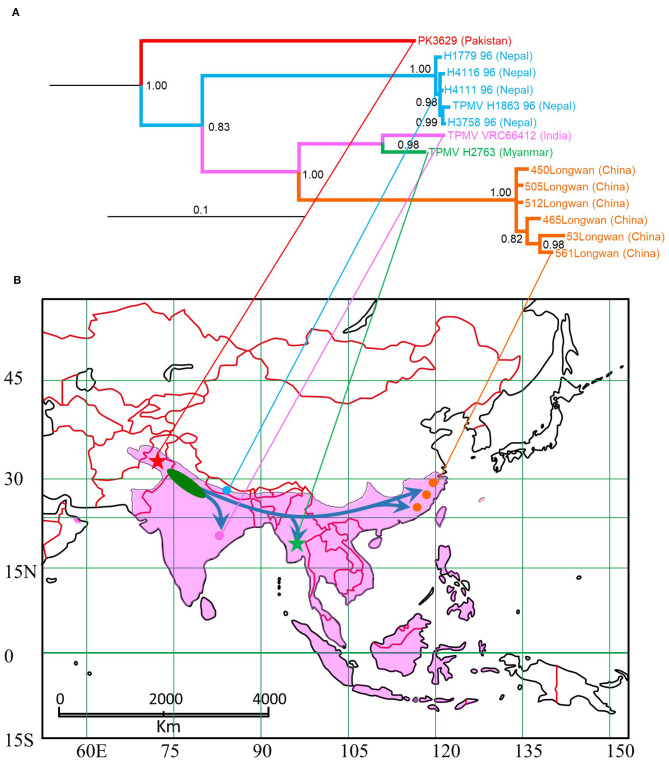
Map of distribution of Asian house shrew and phylogenetic tree based on L segment of TPMV. **(A)** TPMV cluster based on L-segment in phylogenetic analysis. **(B)** Blue arrows in map were estimated expansion root based on TPMV phylogeny. Pakistan strain was captured at red star, Nepal strains (Kang et al., 2011c) were captured at light blue circle, Indian strain (Carey et al., 1971) was captured at pink circle, Myanmar strain was captured at green star and Chinese strains (Guo et al., 2011) were captured at red circles. Pakistan and Myanmar strains were collected in this study (star symbols). Pink area is shown Asian house shrew distribution (Ohdachi et al., 2016).

Although genetically diverse strains of TPMV have been detected in Asian house shrews from Nepal (Kang et al., 2011c) and China (Guo et al., 2011), the geographic distribution and evolutionary origins of TPMV are still unclear. Our data suggest the possibility that TPMV expanded from the Indian subcontinent (Figure 6). The evolutionary time scale of TPMV is faster than that of its host and the host is older than the ancient trade routes between the Middle East and China.

## Conclusion

Disappointingly, TPMV was detected in lung tissues of only two Asian house shrews, one from Myanmar and one from Pakistan. The reasons for this are not entirely clear, but it might be the result of the focal nature of TPMV infection, as is typical of other hantaviruses. Future studies on the phylogeography of TPMV and the Asian house shrew should provide valuable insights into the geographic radiation.

## Data Availability Statement

The original contributions presented in the study are publicly available. This data can be found at: https://www.ncbi.nlm.nih.gov/, with the following accession numbers: MT225396—MT225399; MT344729—MT344941; MT363682—MT363701; MT364899.

## Ethics Statement

The animal study was reviewed and approved by National Institute of Infectious Diseases (NIID), Institutional Animal Care and Use Committee.

## Author Contributions

FK, KA, SM, RY, and SA conceived the study and designed the experiments. SO, KT-T, MM, TJ, NS, SB, KL, TT, CG, MR, HO, IM, HS, and SA conducted the trapping and field collections. FK, KA, and SA performed the experiments. FK, KA, RY, and SA analyzed the data. SO, KT-T, MM, TJ, and NS analyzed the host morphology. FK, KA, KT-T, SM, TM, MS, RY, and SA contributed reagents, materials, and analysis tools. FK, SM, TM, RY, and SA prepared the figures and draft manuscript. All authors contributed to the final manuscript.

## Conflict of Interest

KT, who is deceased, was previously employed by the Applied Biology Co., Ltd., in Tokyo, Japan. The remaining authors declare that the research was conducted in the absence of any commercial or financial relationships that could be construed as a potential conflict of interest.
